# p38 MAPK signaling and phosphorylations in the BRCT1 domain regulate XRCC1 recruitment to sites of DNA damage

**DOI:** 10.1038/s41598-017-06770-3

**Published:** 2017-07-24

**Authors:** Mirta Mittelstedt Leal de Sousa, Karine Øian Bjørås, Audun Hanssen-Bauer, Karin Solvang-Garten, Marit Otterlei

**Affiliations:** 1 Department of Cancer Research and Molecular Medicine, Faculty of Medicine, Norwegian University of Science and Technology (NTNU), Trondheim, Norway; 2 The Proteomics and Metabolomics Core Facility (PROMEC) at NTNU, Trondheim, Norway; 3 The Central Norway Regional Health Authority, Stjørdal, Norway

## Abstract

XRCC1 is a scaffold protein involved in base excision repair and single strand break repair. It is a phosphoprotein that contains more than 45 phosphorylation sites, however only a few of these have been characterized and connected to specific kinases and functions. Mitogen activated protein kinases (MAPK) are mediators of cellular stress responses, and here we demonstrate that p38 MAPK signaling is involved in phosphorylation of XRCC1 and regulation of recruitment to oxidative stress. Inhibition of p38 MAPK caused a marked pI shift of XRCC1 towards a less phosphorylated state. Inhibition of p38 also increased the immediate accumulation of XRCC1 at site of DNA damage in a poly(ADP)-ribose (PAR) dependent manner. These results suggest a link between PARylation, p38 signaling and XRCC1 recruitment to DNA damage. Additionally, we characterized two phosphorylation sites, T358 and T367, located within, or close to, the phosphate-binding pocket of XRCC1, which is important for interaction with PAR. Mutation of these sites impairs recruitment of XRCC1 to DNA damage and binding to PARP1/PAR. Collectively, our data suggest that phosphorylation of T358 and T367 and p38 signaling are important for proper regulation of XRCC1 recruitment to DNA damage and thereby avoidance of potential toxic and mutagenic BER-intermediates.

## Introduction

Our genome is constantly exposed to endogenous and exogenous DNA damaging agents. Reactive oxygen species (ROS) are DNA damaging agents generated as a product of ionizing and ultraviolet radiation, but also through normal cellular metabolism. ROS generate base lesions, apurinic/apyrimidinic (AP) sites and strand breaks that are potentially mutagenic and harmful to cells. X-ray cross-complementing protein 1 (XRCC1) is a scaffold protein and one of the main players in the repair of ROS induced lesions as it binds to and recruits DNA repair proteins to the site of damage^1^.

XRCC1 is rapidly recruited to sites of oxidative damage and single-stranded breaks (SSBs) and the recruitment requires poly(ADP-ribose) (PAR) synthesis by PARP1 or PARP2^2, 3^. The PAR-binding site of XRCC1 has been identified to be the phosphate-binding pocket in the BRCT1 domain^4, 5^. Upon DNA damage, PARP1 binds to the strand break and is thereby activated, triggering the addition of branched PAR-chains on itself and neighboring proteins. This event recruits XRCC1 and other DNA damage response proteins^6^. After recruitment of DNA repair proteins, PAR-chains are rapidly hydrolyzed by PARG, leaving mono(ADP-ribose) (MAR) on the substrates^7^. During PAR hydrolysis, PARP1 is ubiquitinated by the PAR dependent E3 ubiquitin ligase RNF146, leading to degradation of PARP1^8^. XRCC1 is retained at the DNA damage site through binding of the BRCT2 domain^9^.

After DNA damage detection, DNA damage signaling is induced. PARP1 is a sensor of DNA single strand breaks (SSBs), however much remains unknown about the signaling downstream of PARP1; which kinases are involved and how these events affect XRCC1 recruitment to DNA damage. ATM (Ataxia-telangiectasia mutated), ATR (ATM- and Rad3-Related) and DNA-PK (DNA-dependent protein kinase) are members of the phosphatidylinositol-3-kinase like kinase family (PIKKs) and are the upstream kinases in the DNA damage response. Upon activation, they phosphorylate a wide range of substrates. ATR become activated by RPA bound to ssDNA during replication stress, while ATM and DNA-PK are activated at DSBs by the MRN-complex and Ku70/Ku80, respectively^10^. However, there have been reports indicating that PARylation can activate PIKKs. PARP1 and the DNA-dependent protein kinase (DNA-PK) are substrates of each other and DNA-PK auto phosphorylation is stimulated by PARP1 mediated PARylation^11^. Additionally, PARylation dependent activation of ATM has been demonstrated^12^. The p38 mitogen activated protein kinase (MAPK) pathway has also been linked to the DNA damage response. It has been demonstrated that p38 signaling leads to G2/M-arrest in response to UV and ROS^13, 14^ and that this is independent of ATM/ATR signaling^15, 16^. In addition to DNA damage signaling and DNA repair, both PARP1 and p38 play a role in regulation of the immune response and both are therefore explored as promising drug targets for inflammatory diseases, as well as cancer (reviewed in refs 17 and 18). How inhibition of these affects DNA repair is therefore of clinical interest.

XRCC1 is known to be an extensively phosphorylated protein with more than 45 known phosphorylation sites, mainly found in the region between the N-terminal and BRCT1 domain, and the inter-BRCT region^19^. The latter contains at least six sites that show attenuated phosphorylation when the CK2 isoforms α and α’ are knocked down. These 7 sites are necessary for XRCC1’s interaction with the FHA domains and activity of the end-trimming enzymes such as polynucleotide kinase (PNK), aprataxin (APTX) and PNK-like factor (APLF)^20–23^. DNA-PK phosphorylates S371 in the BRCT1 domain, an event suggested to cause XRCC1-homodimer dissociation^24^. Furthermore, the protein kinase Chk2, a substrate of ATM, has been suggested to phosphorylate T284 and possibly increase the interaction with DNA glycosylases^25^. However, no direct links between kinases and recruitment of XRCC1 have been established. Here we demonstrate that p38 signaling in a PAR-dependent manner, indirectly or directly is involved in moderating the recruitment of XRCC1 to sites of DNA damage. Furthermore, p38 signaling affects the phosphorylation state and capacity of XRCC1 to support DNA repair. Importantly, we identify two phosphorylation sites within the BRCT1 domain, T358 and T367, which is important for both recruitment of XRCC1 to DNA damage and for PAR-binding. These two sites are located within the phosphate-binding pocket of XRCC1.

## Results and Discussion

### p38 inhibition changes the global phosphorylation state of XRCC1

**Figure 1:**
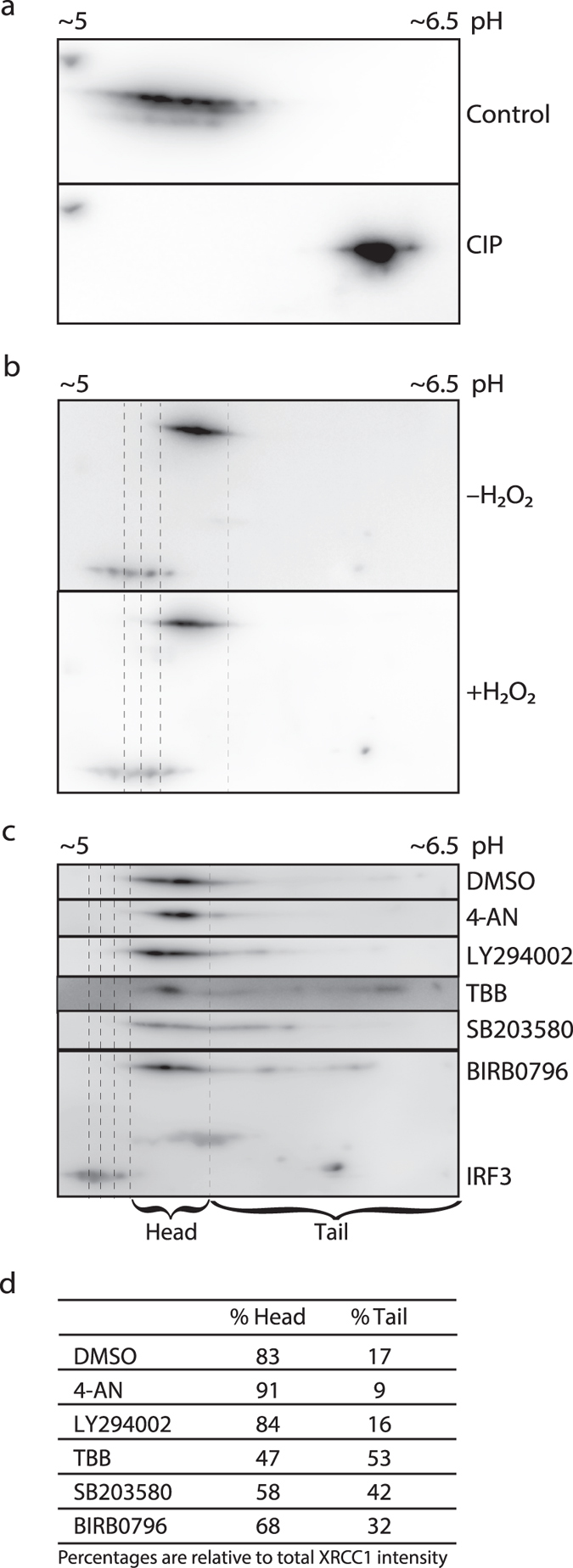
Phosphorylation state of endogenous XRCC1. (**a**) 2D-PAGE western analysis of immunoprecipitated endogenous XRCC1, mock treated (control) and calf-intestine phosphatase (CIP) treated immunoprecipitates. (**b**) 2D-PAGE western analysis of whole cell lysates from HeLa cells grown in parallel with and without addition of H_2_O_2_ (62.5 µM). (**c**) 2D-PAGE western analysis of whole cell lysates from HeLa cells treated with either 0.25% DMSO (mock), 10 µM 4-AN (PARP inhibitor), 25 µM LY294002 (PI3K inhibitor), 25 µM TBB (CK2 inhibitor), 25 µM SB203580 (p38 MAPK inhibitor), or 25 µM BIRB0796 (p38 MAPK inhibitor). Recombinant IRF3 (Interferon regulatory factor 3) was added to all the lysates to serve as an internal positional reference during picture alignment (dotted lines). IRF3 is depicted only in the 2D-PAGE western analysis of BIRB0796 treated cells for simplicity. Cropped images of one out of two gels with similar patterns is depicted. (**d**) Quantification of %Head and %Tail (defined in C) using the Kodak Molecular Imaging software version 4.0.1. Ratios of “head” and “tail” densities versus total XRCC1 intensity (head + tail) were calculated after subtracting background intensity values.

There are more than 45 reported phosphorylation sites in XRCC1, but only a fraction of these has been characterized. We examined the phosphorylation state of XRCC1 in response to selected kinase inhibitors, a PARP inhibitor and hydrogen peroxide (H_2_O_2_) treatment, in order to assess the impact of potential kinases, PARylation and oxidative stress, respectively. Using two-dimensional gel electrophoresis combined with western blot analysis we found that endogenous XRCC1 shifted towards a more alkaline pI when treated with alkaline phosphatase, confirming that XRCC1 is naturally present in a multi-phosphorylated state (Fig. 1a, uncropped images in Supplementary Fig. S1)^26^. Treatment with H_2_O_2_ did not substantially change the overall phosphorylation state of XRCC1 (Fig. 1b). Similarly, treatments with a PI3K inhibitor (LY294002) or a PARP inhibitor (4-AN) did not cause any major phosphorylation shifts, i.e. an increase in “tail” (Fig. 1c, quantification of % protein in “tail” and “head” are given in Fig. 1d). In contrast, inhibition of CK2 (TBB) resulted in a considerable shift of XRCC1 towards a less phosphorylated state (Fig. 1c,d). This was expected since XRCC1 contains at least six CK2 phosphorylation sites^20, 21^. Interestingly, a clear visible shift towards a less phosphorylated state was also detected after inhibition of p38 (SB203580) (Fig. 1c,d). To verify that p38 is the main target causing these effects, the experiment was repeated using the structurally unrelated p38 inhibitor BIRB0796, as recommended^27^. BIRB0796 treatment also caused a marked shift in pI (Fig. 1c,d), confirming that p38, directly or indirectly via downstream activation of phosphatases and kinases, is involved in regulating the phosphorylation status of XRCC1.

### Inhibition of p38 MAPK increases XRCC1 recruitment to sites of DNA damage

**Figure 2:**
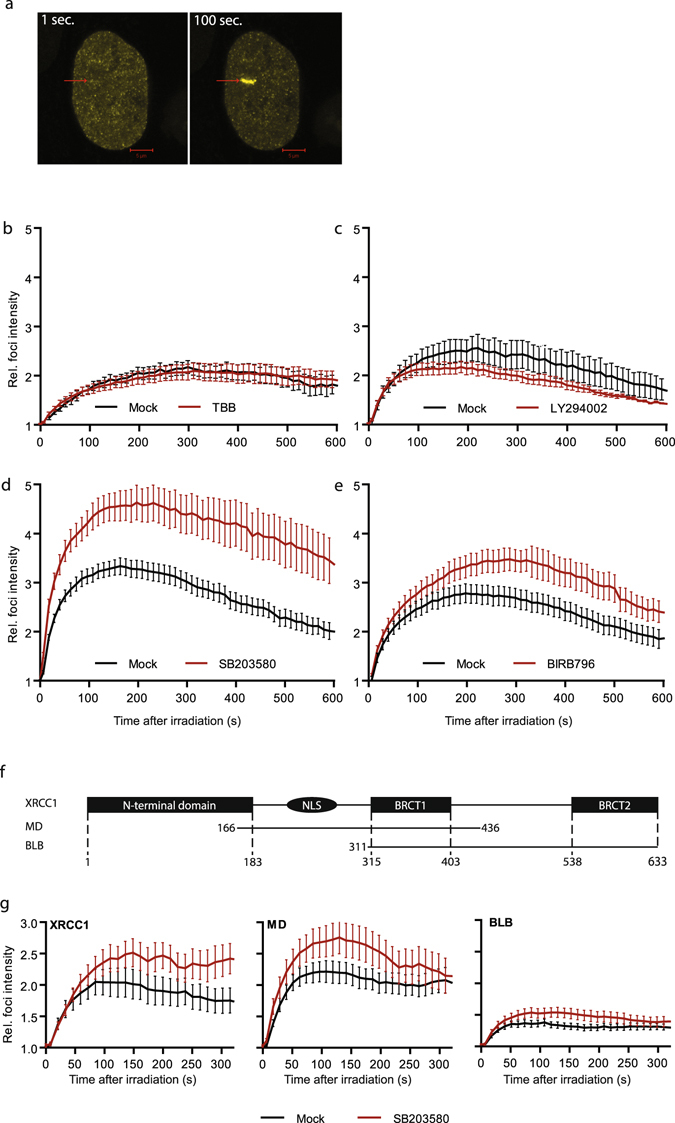
Recruitment of XRCC1-YFP and deletion mutants to micro-irradiated regions after kinase inhibitor treatment. (**a**) Untreated, freely cycling cells stably expressing XRCC1-YFP 1 s and 100 s after 405 nm micro-irradiation in a selected region (marked with arrow). Bars 5 µM. (**b–e**) Average fold increase in mIF intensities of XRCC1-YFP in HeLa cells treated with either 0.25% DMSO (mock, black lines) or kinase inhibitors (red lines). Mean ± SEM is shown. (**b**) 25 µM CK2 inhibitor TBB. Mock n = 4, TBB n = 9. **(c)** 25 µM PI3K inhibitor LY294002. Mock n = 16, LY294002 n = 7. (**d**) 25 µM p38 MAPK inhibitor SB203580. Mock n = 12, SB203580 n = 12. (**e**) 25 µM p38 MAPK inhibitor BIRB0796. Mock n = 11, BIRB0796 n = 16 (**f**) Schematic overview of full length XRCC1 and deletion mutants. (**g**) Average fold increase in mIF intensities of full-length XRCC1-YFP (left panel), YFP-MD (middle panel) and YFP-BLB (right panel) deletion mutants in CHO EM9 cells in absence (black line) and presence of 25 µM SB203580 (red line). The graphs representing the mutants in absence of SB203580 have previously been published^29^. XRCC1: Mock n = 15, SB203580 n = 11. MD: Mock n = 15. SB203580 n = 16. BLB: Mock n = 20, SB203580 n = 17.

The change in phosphorylation status of endogenous XRCC1 after kinase inhibitor treatment prompted us to investigate the effect of these inhibitors on recruitment of a fluorescently tagged XRCC1 construct to DNA damage sites exposed to 405 nm UVA micro-irradiation. In the following experiments, XRCC1-YFP expressing cells were irradiated with a low UV dose (1x dose, see materials and methods) that does not induce DSBs and accumulation of γH2AX, but induces ROS and causes SSBs and base damage^28^. Accumulation of XRCC1 in the irradiated area is shown in Fig. 2a. Inhibition of CK2 or PI3K had no, or minor, effects on XRCC1 accumulation at micro-irradiation foci (mIF) (Fig. 2b,c). Interestingly, inhibition of p38 by both SB203580 and BIRB0796, markedly increased XRCC1 accumulation at mIF (Fig. 2d,e). This suggests a role of p38 in the regulation of XRCC1 recruitment to ROS induced DNA damage.

To narrow down the regions for possible phosphorylation sites affected by p38 signaling, we tested the response of p38 inhibition on two deletion mutants of XRCC1, MD and BLB, both which contain the BRCT1 domain (Fig. 2f) and are recruited to mIF^29^. The MD mutant and full-length XRCC1 had a similar increase in recruitment to mIF, while the BLB mutant displayed a reduced accumulation. Still, an increase in accumulation after p38 inhibition was observed also for BLB (Fig. 2g). This suggests that phosphorylation sites in the BRCT1 region, that is common for both MD and BLB, might be directly or indirectly affected by p38 signaling. In order to avoid the many phosphorylation sites in the inter-BRCT1 domain, we used the MD deletion mutant for the following experiments.

### p38 signaling regulates the kinetics of repair and is dependent on PARP1 activity

**Figure 3:**
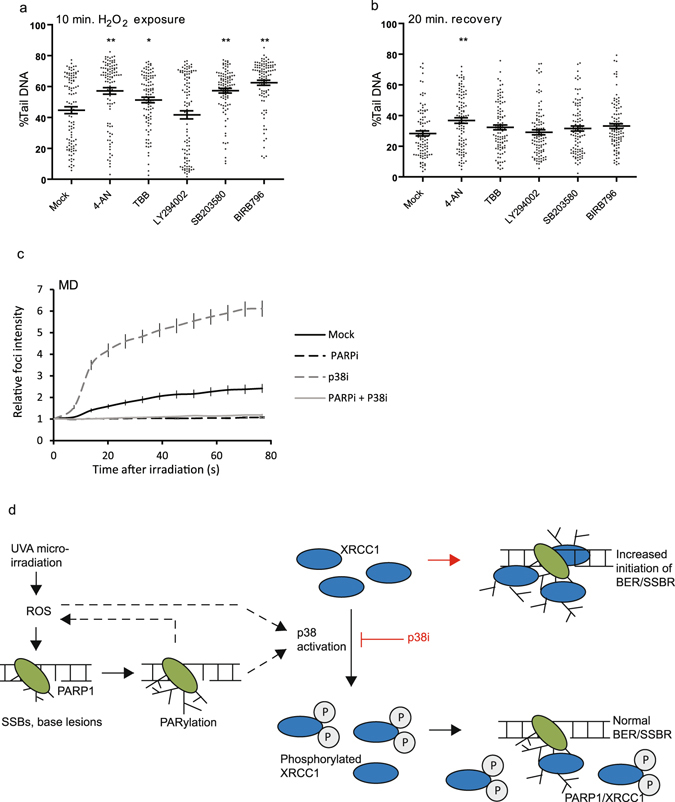
Effect of PARP- and kinase inhibitors on repair and recruitment to DNA damage. (**a**,**b**) Comet analysis of PARylation- and kinase inhibitor treated cells harvested after 10 minutes of H_2_O_2_ insult (47 µM) (**a**) and after 20 min recovery (**b**). Dot plot of cells treated with 0.25% DMSO (mock), 10 µM 4-AN (PARPi), 25 µM TBB (CK2i), 25 µM LY294002 (PI3Ki), 25 µM SB203580 (p38i), or 25 µM BIRB0796 (p38i). Averages of n = 100 ± SEM. One out of four similar experiments is shown. Asterisks represent Student’s t-test p-values from the corresponding kinase/PARylation inhibitor treated vs. mock treated samples, where **p < 0.001, *p < 0.05. (**c**) Average fold increase in mIF intensities of YFP-MD in cells treated with either 0.25% DMSO (Mock), 10 µM SB203580 (p38i) and/or 10 µM PJ34 (PARPi) at a 1x laser dose. Mock: n = 11, p38i: n = 11, PARPi: n = 5, PARPi + P38i: n = 6. (**d**) Model of PAR and p38 signaling in recruitment and repair of SSBs and base lesions. Dashed arrows illustrate possible ways of p38 activation. Red arrow and line indicate preferred pathway in presence of p38i.

To assess whether the accumulation of XRCC1 at sites of DNA damage after p38 inhibition affected XRCC1’s ability to support BER/SSBR, we performed Comet assays on cells treated with inhibitors and H_2_O_2_. Inhibition of PI3Ks (LY294002), used as a negative control in this setting as it did not affect recruitment of XRCC1, did not alter the average genomic fragmentation compared to mock treated cells (Fig. 3a). p38 inhibition (SB203580 and BIRB0796), on the other hand, resulted in a significant increase in initial genomic fragmentation. This was also observed for inhibition of PARylation (4-AN) and CK2 (TBB) (Fig. 3a), in accordance with previous reports^20, 21, 30^. No increase in genomic fragmentation compared to mock treated controls was observed in inhibitor treated cells in absence of H_2_O_2_ (data not shown). After 20 min recovery, only the cells treated with PARP inhibitor (4-AN) had increased levels of strand breaks compared to control (Fig. 3b). This suggests that increased XRCC1 accumulation leads to increased levels of AP sites or strand breaks in regions of oxidative DNA damage. Lesions and repair-complex-DNA intermediates that occur in close proximity in different strands will appear as strand breaks in the Comet assay. Repair intermediates such as AP sites and strand breaks are potentially toxic and mutagenic, thus tight regulation of BER/SSBR is important. Notably, increased accumulation of XRCC1 at sites of DNA damage and increased transient DNA fragmentation after inhibition of p38 may represent undesired effects that should be considered when using p38 inhibitors as anti-inflammatory drugs.

Efficient XRCC1 recruitment to UVA induced damage sites is dependent on PARP1 and PARylation^2, 3^, thus we examined whether the increased recruitment after p38 inhibition was dependent on PARylation. We found that inhibition of PARP1 with PJ34, which does not trap PARP1 to site of DNA damage^28^, abolished the recruitment of MD to mIF. Furthermore, inhibition of p38 did not increase the recruitment in absence of PARylation (Fig. 3c). In support of a link between PARylation and activation of p38 signaling, it has been reported that p38 activation is impaired in PARP1 deficient cells^31, 32^. Additionally, PARP1 activity by itself increases ROS production, which may contribute to amplification of ROS mediated p38 activation^33, 34^.

In summary, our data demonstrate that XRCC1 recruitment to mIF is dependent on PARylation and modulated by p38 signaling. However, the exact connection between p38 signaling and PARylation remains elusive. We hypothesize that PARylation at SSBs is involved in activation of p38. Our data demonstrate that p38 mediated signaling regulates the phosphorylation status of XRCC1, and thereby its recruitment to DNA damage. When phosphorylation of XRCC1 is reduced, more XRCC1 is recruited to sites of DNA damage, leading to a transient increase in AP sites and strand breaks (Model illustrated in Fig. 3d).

### Mutations in XRCC1 phosphorylation sites in the BRCT1 domain impair recruitment to DNA damage

**Figure 4:**
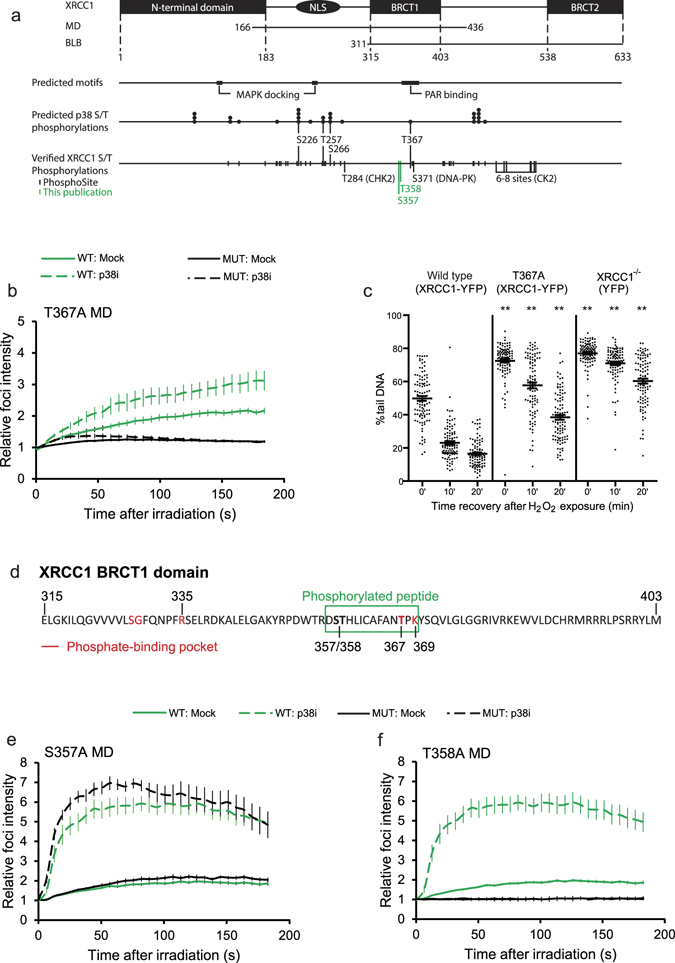
Mutation of XRCC1 phosphorylation sites and recruitment to DNA damage. (**a**) Map of XRCC1: known domains, deletion mutants, predicted MAPK docking motif, PAR-binding region, predicted p38 MAPK and known phosphorylation residues. Phosphorylation residues identified in this publication is marked in green. (**b**) Average fold increase in mIF intensities of wild type YFP-MD (WT, green lines) and mutant YFP-MD T367A (MUT, black lines) in mock and p38i (SB203580, 25 µM) treated CHO EM9 cells using 1x laser dose. WT: Mock n = 19, p38i n = 12. M﻿UT (﻿T367A): Mock n = 17, p38i n = 16. Mean ± SEM. (**c**) Comet analysis: Dot plot of CHO EM9 cells expressing XRCC1-YFP wild type, XRCC1-YFP T367A and YFP treated with H_2_O_2_ (62 µM) for 10 min. 0, 10 and 20 min recovery. Averages of n = 100 ± SEM. One out of three similar experiments is shown. Asterisks represent Student’s t-test p-values from cells with mutated or no XRCC1 vs. the corresponding XRCC1 wild type sample, where **p < 0.001. (**d**) Sequence of the XRCC1 BRCT1 domain. The phosphorylated peptide detected on MS (green box) and the phosphate-binding pocket (red) are indicated. (**e**,**f**) Average mIF intensities of wild type YFP-MD (WT, green lines) and YFP-MD T357A (**e**) and T358A (**f**) mutants (MUT, black lines) in mock and p38i (SB203580, 25 µM) treated HeLa cells using 1x laser dose. WT: Mock n = 16, p38i n = 10. S357A: Mock n = 16, p38i n = 11. T358A: Mock n = 8, p38i n = 4. Mean ± SEM.

We performed a search for predicted p38 as well as general MAPK phosphorylation sites in XRCC1 *in silico* using five different prediction algorithms. From the predicted sites (see Fig. 4a), we selected putative phosphorylation sites for serine/threonine (S/T) to alanine (A) mutagenesis based on the following criteria: 1) suggested by more than two prediction algorithms 2) reported to be phosphorylated 3) within MD (aa 166–436). The candidate phosphorylation sites fulfilling these criteria were S226, T257 and S266 (Fig. 4a). Considering the importance of the BRCTI domain in recruitment of XRCC1 to sites of DNA damage^5, 29^ we also included T367. This is the only predicted MAPK site within the BRCT1 domain and it was recently shown to be phosphorylated^35^ and part of the phosphate-binding pocket^5^. However, the functional role of phosphorylation at T367 is not known.

Micro-irradiation experiments showed that the S226A, T257A, and S266A mutants responded to p38 inhibition similarly to wild type (Supplementary Fig. S2). Phosphorylations at T257 and S266 have recently been suggested to be involved in nuclear import and binding to Importin α^36^, however we did not observe any change in nuclear localization of the single mutants. Mutation of T367 on the other hand, almost completely abrogated recruitment of MD to mIF. Inhibition of p38 induced a low and transient increase in recruitment (Fig. 4b), suggesting that T367 could be one, but not the only target of p38 signaling. Similar results were obtained for full-length XRCC1 T367A (data not shown). XRCC1 deficient cells complemented with full-length XRCC1 T367A accumulated almost as much genomic fragmentation as XRCC1 deficient cells after oxidative stress (Fig. 4c). XRCC1 T367A cells had an increased repair rate compared to XRCC1 deficient cells, but a slower repair rate than wild type XRCC1 complemented cells (Fig. 4c). This indicates that the attenuated recruitment of XRCC1 T367A to DNA damage sites caused a reduced rate of DNA repair.

Next, we analyzed XRCC1 isolated from cells treated with H_2_O_2_ and/or p38 inhibitor by mass spectrometry (MS), in order to detect possible dynamic changes of T367 phosphorylation in response p38 inhibition and oxidative stress. We were able to identify phosphorylated forms of the peptide containing T367 (Fig. 4d). However, the phosphorylations of this peptide were assigned to two other sites, S357 and T358, not previously reported to be phosphorylated (Supplementary Fig. S3). We could not determine changes in phosphorylation of these sites upon p38 inhibition and oxidative stress, possibly due to low levels of the phosphorylated peptide.

The three phosphorylation sites S357, T358 and T367, are all within or close to the phosphate-binding pocket of XRCC1 important for interaction with PAR^4, 5^ (Fig. 4d). Mutations of R335 and K369 were recently shown to impair XRCC1 interaction with PAR, and thereby recruitment of XRCC1 to sites of DNA damage^5^. Therefore, we next investigated whether mutations of the two novel phosphorylation sites identified, S357 and T358, affected recruitment of MD to mIF. The mutant S357A was similarly recruited as wild type MD, while the T358A mutation abolished accumulation in mIF (Fig. 4e,f). Inhibition of p38 did not induce any accumulation of T358A (Fig. 4f). Using four different algorithms for prediction of kinase consensus sites we found that all three sites, S357, T358 and T367, could be phosphorylated by MAPKs or substrates of p38 MAPK (Table 1). Among several other potential kinases, one algorithm proposed the central DDR kinase DNA-PK as a candidate for all three residues. Together, these results suggest that the T358 residue, in addition to T367, could be targets of p38 signaling.

**Table 1:** Kinase predictions of the phosphorylated residues S357, T358 and T367. Kinases involved in the MAPK pathway or p38 substrates are bolded and kinases involved in DDR are marked in grey.

*Resource*	*S357*	*T358*	*T367*
ELM^42^	**GSK3**	**GSK3**	**GSK3**
	PKA	PKA	**MAPK**
NetworKIN^43^	DNA-PK	DNA-PK	DNA-PK
NetPhosK^44^	PKA		**GSK3**
GPS^45^	DMPK	CIT	CDK2
	GRK	DYRK2	CDK5
	RPS6KA4	TTK	CDK9
	CK1	STK39	**ERK1**
	**MNK2 (MAPK signal-integrating kinase 2)**	**TAK1 (MAPK3K7)**	**ERK2**
	VRK2		**ERK4**
	AURKC		**MAPK12/p38 gamma**
			IRAK

**Figure 5:**
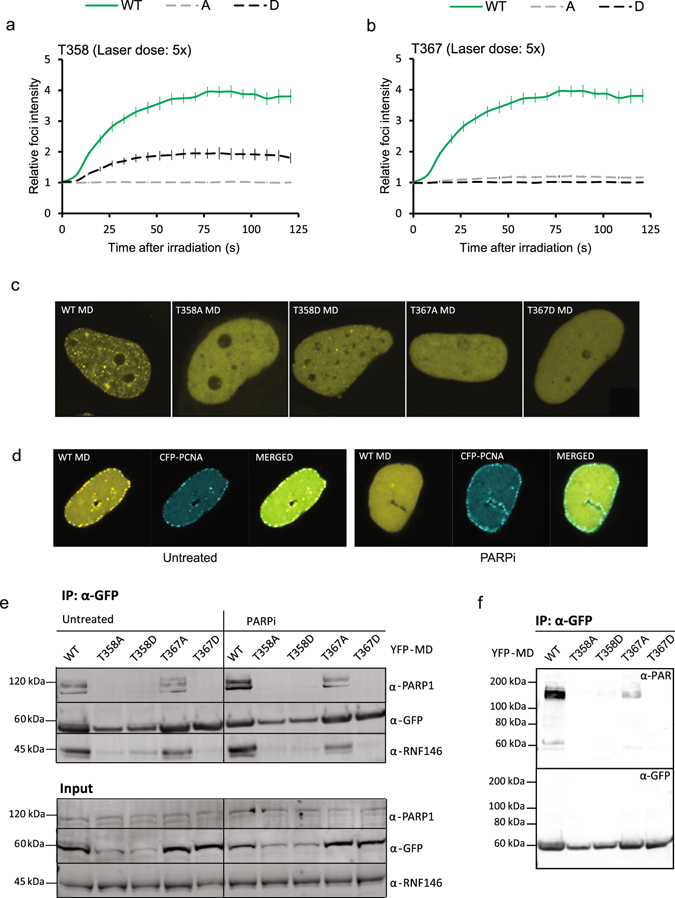
PAR-binding and recruitment to DNA damage of phosho-mimetic and phospho-defective T358 and T367 MD mutants. Average fold increase in mIF intensities of YFP-MD phospho-mimetic and phospho-defective mutants (**a**) T358 and (**b**) T367 using a 5x laser dose. Wild type YFP-MD (WT, green line): n = 10, T358A (grey dotted line): n = 8, T358D (black dotted line): n = 10, T367A (grey dotted line): n = 10, T367D (black dotted line): n = 8. Mean ± SEM. (**c**) Intracellular localization of wild type (WT), T358A/D, T367A/D YFP-MD in HeLa cells. (**d**) Intracellular localization of wild type YFP-MD and CFP-PCNA in CHO EM9 with and without PARPi (PJ34, 10 μM, 1 hour). (**e**) Co-immunoprecipitation of PARP1 and RNF146 with wild type, T358A/D and T367A/D YFP-MD with and without PARPi (PJ34, 10 μM, 1 hour) using 100 mM wash buffer. Cropped images of one out of five experiments with similar trends are depicted. (**f**) Co-immunoprecipitation of PARylated proteins with wild type, T358A/D and T367A/D YFP-MD using 1 M wash buffer. One out of three experiments with similar trend is depicted.

To explore the function of the two phosphorylation sites T358 and T367 further, we created phospho-mimetic aspartate (D) mutants and analyzed recruitment to mIF. Both A and D mutants had strongly reduced ability to accumulate at site of DNA damage compared to wild type even at a high laser dose (5x) (Fig. 5a,b). However, T358D was clearly recruited whereas minor accumulation was observed for T367A. Inhibition of p38 increased recruitment of T358D similarly to T367A (data not shown and Fig. 4b), suggesting that none of these are the only target of p38 signaling. Although all mutants are localized to nuclei, they contained variable amounts of nuclear foci. Wild type MD appeared in nuclear foci in most cells (Fig. 5c). These foci represent both stress induced foci and replication foci^29^. In contrast, the MD mutants did not appear in nuclear foci, with exception of T358D that localized to nuclear foci in some cells, but at a reduced strength compared to wild type (Fig. 5c). All MD wild type foci, also those colocalizing with PCNA and thus representing replication foci, disappeared after treatment with PARP inhibitor (Fig. 5d). When we examined the MD phospho-mutants’ ability to colocalize with PCNA in replication foci they all colocalized to some extent in XRCC1 deficient cells (CHO EM9), but less or not at all in HeLa cells (Supplementary Fig. S4). This could indicate that the mutants are to some degree outcompeted by endogenous XRCC1 in HeLa cells, which could be explained by reduced affinity for PARylated proteins.

Hence, we next examined the PAR- and/or PARP1-binding properties of the MD mutants by immunoprecipitations in presence and absence of two PARP inhibitors. Immunoblotting revealed that higher levels of PARP1 were pulled down with wild type MD compared to all mutants. T367A pulled down some PARP1, while no detectable PARP1 was pulled down with T367D and T358A/D (Fig. 5e, Supplementary Fig. S5a,b). We also examined the levels of the PAR dependent E3 ubiquitin ligase RNF146, known to target PARP1 and several other repair proteins including XRCC1 for degradation^8^. The levels of RNF146 in the pull down followed the same pattern as PARP1 (Fig. 5e, Supplementary Fig. S5a,c). Interestingly, wild type MD pulled down more PARP1 and RNF146 from cells treated with PARP inhibitor (Fig. 5e, Supplementary Fig. S5b,c). This was observed using both PJ34 and 4-AN (data not shown) at doses that abolish nuclear PARylation^28^. These results deviate from previously published pull down experiments using a MD like domain of XRCC1 (aa 161–406) and the PARP inhibitor KU58948^5^. The reason for this is not known, but it could be due to differences in the PARP inhibitors ability to interfere with PARP1 binding to chromatin. PARP1 trapping can be a problem in such studies and has been suggested for several PARP inhibitors (4-AN, niraparib, olaparib and veliparib)^28, 37^.

To further examine the PAR-binding properties of the MD mutants we washed immunoprecipitates in high salt buffer (1 M) to remove low affinity binders. All mutants pulled down less PARylated proteins compared to wild type, only a few weak bands were detected in the T367A and T358D pull downs (Fig. 5f, quantification in Supplementary Fig. S5d). These data support that phosphorylation of these residues are important for regulating the affinity to PARylated proteins.

In summary, the results presented here demonstrate a role of p38 signaling and phosphorylations in the BRCT1 domain of XRCC1 for accumulation at sites of DNA damage as well as sites of replication. The p38 signaling pathway has been extensively studied as mediator of cell cycle regulation and apoptosis in response to ROS, UV-irradiation, and/or DNA-lesions (reviewed in ref. 38), but this is to our knowledge the first time it has been shown to influence the dynamics of recruitment of DNA repair proteins. 2D-PAGE analysis demonstrated that p38 clearly alters the phosphorylation status of XRCC1. However, XRCC1 is not necessarily a direct substrate of p38. We show that T367 in XRCC1, an *in silico* predicted p38 phosphorylation site, is a functional phosphorylation site that has importance for binding to PAR and thereby recruitment to sites of DNA damage. We also identify T358 as a novel functional phosphorylation residue that affects the affinity to PAR. These results demonstrate that phosphorylations in or around the phosphate-binding pocket are important for a dynamic regulation of affinity to PAR and thereby recruitment to DNA damage.

## Methods

### Fluorescent tagged protein constructs

pXRCC1-YFP and the XRCC1 deletion mutants pYFP-XNTD, pYFP-MD, and pYFP-BLB have been previously described^28, 39, 40^.

### Cell lines

HeLa S3 and CHO EM9 cells (ATCC) were cultured in DMEM (Sigma-Aldrich) and alpha-modified MEM (Sigma-Aldrich) respectively. Both media were supplemented with 10% FCS (GIBCO), 250 µg/ml amphotericin B (Sigma-Aldrich), 100 µg/ml gentamicine (Invitrogen), and 1 mM L-glutamine (Bio Whittaker). The cells were cultured at 37 °C in a 5% CO_2_ humidified atmosphere. CHO EM9 stably expressing pXRCC1-YFP was made by prolonged culturing in medium containing 400 μg/ml geneticine (G418; Invitrogen), cell sorting (BD FACSAria), and cloning by dilution.

### Preparation of cell lysates

Exponentially growing HeLa S3 cells were treated 1 hour at 37 °C with 0.25% DMSO (mock), 10 µM 4-amino-1,8-naphthalimid (4-AN) (Sigma-Aldrich), or 25 µM of a kinase inhibitor; TBB (Sigma-Aldrich), LY294002 (Sigma-Aldrich), SB203580 (Sigma-Aldrich), BIRB0796 (Axon Medchem). The cells were stressed 10 minutes with 62.5 µM H_2_O_2_ and subsequently detached using 600 µM EDTA in PBS, harvested in ice cold 10% FCS in PBS, and pelleted by centrifugation. Whole cell lysates were prepared by carefully resuspending the cell pellet in 3x packed cell volume in buffer I; 20 mM pH 7.8 HEPES-KOH, 100 mM KCl, 1.5 mM MgCl_2_, 0.2 mM EDTA, 20% glycerol, 0.5% NP-40, 1 mM DTT, 1x complete protease inhibitor, and 5x phosphatase-inhibitor cocktails I and II (Sigma-Aldrich). 400 U Omnicleave endonuclease (Epicenter Technologies) was added to each vial and the resuspended cells sonicated (Branson Sonifier 250). After sonication residual DNA/RNA in the lysates were digested 1 hour at 37 °C using a endonuclease cocktail of 400 U Omnicleave, 10 U DNase I (Roche Inc.), 250 U benzonase (EMD), 100–300 U micrococcal nuclease (Sigma-Aldrich), and 20 µg RNase (Sigma-Aldrich) per 30 mg protein in the lysate. Digestion was followed by clearance by centrifugation.

### 2D-SDS PAGE

To visualize the phosphorylation pattern of XRCC1, 200 μg of protein lysates from kinase inhibited HeLa S3 cells were separated by two-dimensional polyacrylamine gel electrophoresis (2D-PAGE) and visualized by western blot. 40 ng of recombinant Interferon regulatory factor 3 (IRF-3) (pI 5.17, MW 47.2 kDa) was added to each sample as an internal localization standard to monitor differential XRCC1 migration. 2D-PAGE was performed using Immobiline DryStrips pH 4–7 (GE Healthcare) and pre-cast 4–12% denaturing NuPAGE gels (Invitrogen). Western blot analysis was performed using mouse monoclonal XRCC1 antibody (ab1838, Abcam) and rabbit polyclonal IRF3 antibody (4962, Cell Signalling) as primary antibodies, followed by HRP conjugated secondary rabbit anti-mouse and swine anti-rabbit antibodies (Dako Denmark). Blots were developed using SuperSignal West Femto Maximum Sensitivity Substrate (Thermo Scientific) and scanned in IS4000R Kodak imager (Fisher Scientific). Quantification of spot intensities was performed by using the Kodak Molecular Imaging software version 4.0.1. After subtracting background intensity values, ratios of densities of “head” and “tail” regions versus total XRCC1 intensity (head + tail) were calculated.

### Alkaline phosphatase treatment

Endogenous XRCC1 from HeLa cells was immunoprecipitated over night at 4 °C using Dynabeads protein A magnetic beads coupled to monoclonal XRCC1 antibodies (ab1838, Abcam). The beads were washed three times with a 10 mM Tris-HCl pH 8 and 50 mM KCl buffer, and divided in two equal parts prior to resuspension in phosphatase buffer (10 mM Tris-HCl pH 7.9, 50 mM NaCl, 10 mM MgCl_2_, 1 mM DTT, and 0.1 mM ZnCl_2_). Excess of calf intestinal phosphatase (CIP, Biolabs) was added to one of the tubes following incubation for 1 hour at 37 °C. Proteins were eluted from beads by overnight incubation in Destreak solution containing 1% IPG buffer pH 4–7 (GE Healthcare). Eluates were collected and submitted to 2D-PAGE as described above.

### Confocal analysis

Analysis was performed using a Zeiss LSM510 Meta laser scanning microscope equipped with a Plan-Apochromate 63x/1.4 oil immersion objective. Live cell images were acquired in medium, with the stage heated to 37 °C. YFP was excited at λ = 514 nm and detected at λ = 530–600 nm, using consecutive scans. The thickness of the scanned optical slices was 1 µm. No image processing except contrast and intensity adjustments was performed.

### 405 nm micro-irradiation

A 405 nm diode laser was focused through a 63x/1.4 Plan-Apochromate oil objective e to a diffraction limited spot size in a Zeiss LSM 510 Meta laser scanning microscope. The 405 nm diode output was measured to 30 mW using a FieldMaster GS energy meter (Coherent Inc.) with a low power probe. Micro-irradiation was performed with various laser doses; the lowest with 60 beam iterations at a speed of 1.27 µsec/pixel over a 50 × 2-pixel area (0.23 µm^2^). Efficient energy applied is approximately 30 pJ/1200 iterations (65 mJ/cm^2^), a dose that induce ROS and SSBs (further referred to as 1x dose). A 15x dose was required to induce DSBs^28^. The dose was increased using higher number of iterations. Time lapse image acquisition started one scan prior to micro-irradiation. Signal intensities were measured using the LSM 510 Meta operating software version 4.2. The relative signal strength of foci was obtained by dividing average foci strength with average signal strength measured in a non-irradiated, equally sized, region of the nucleus. Only cells with similar intensities were analyzed.

### Comet assay (alkaline single cell gel electrophoresis)

HeLa S3 were pre-treated for 1 hour at 37 °C with 0.25% DMSO (mock), 10 µM 4-amino-1,8-naphthaliamid (4-AN), or 25 µM of a kinase inhibitor; TBB, LY294002, SB203580, BIRB0796. These cells and CHO EM9 stably transfected with XRCC1-YFP T367A, XRCC1-YFP or YFP were next exposed to 10 min of 47 or 62 µM H_2_O_2_ (Sigma-Aldrich) in PBS at 37 °C. The cells were washed twice with PBS, harvest or kept in growth medium with the respective inhibitors for further recovery. Cells were harvested in ice cold 30% FCS in PBS and subjected to lysis ON, alkaline DNA unwinding (pH > 13.3), and single cell electrophoresis as described^28^. 100 comets were selected randomly from each slide and evaluated using Komet 5.0 imaging software (Andor Technology).

### XRCC1 map and predictions

Predictions of interaction motifs and p38 phosphorylation sites on XRCC1 were performed by using several different online services. XRCC1 protein sequence was uploaded as FASTA format acquired from UniProt^41^, as reference to its UniProt identifier (Uniprot ID: p18887). If possible all isoforms of p38 were tested, if not the prediction was narrowed down to p38 α (also known as MAPK14). Prediction of MAPK motif was from ELM^42^. Prediction of p38 phosphorylation sites in XRCC1 was performed using the following servers; NetworKIN^43^, NetPhosK 3.1^44^, GPS 2.1^45^, and CRPhos^46^. Nuclear localization signal (NLS) and PAR-binding motifs have been characterized^5, 36^. Known phosphorylation sites of XRCC1 in the map are mainly from global mass spectrometry analysis summarized in PhosphoSite^19^, except phosphorylations performed by DNA-PKcs^24^, CHK2^25^ and CK2^20^.

### Immunoprecipitation

Immunoprecipitations were performed using Dynabeads protein A magnetic beads coupled to polyclonal GFP antibodies (ab290, Abcam) using the crosslinker, Bis(sulfosuccinimidyl)suberate (BS3), according to manufactures instructions (Thermo Scientific). *For MS-analysis*: Coupled beads were incubated with XRCC1-EYFP overexpressed HeLa S3 cell lysates of untreated cells or cells treated with H_2_O_2_ under gentle rotation at 4 °C overnight followed by washing 3 times with 10 mM Tris-HCl pH 7.5, 50 mM KCl before elution. *Co-immunoprecipitation analysis*: Beads were incubated with lysates from HeLa S3 cells transfected with YFP-MD wild type and mutant plasmid construct. The cells were pretreated for 1 hour at 37 °C with 0.25% DMSO (mock) or 10 µM PARP inhibitor (PJ34). Whole cell extracts were prepared as described above, with exception of PJ34, that were added to buffer I to a concentration of 10 µM prior to lysis. Beads were washed three times in 10 mM Tris-HCl pH 7.5 containing either 100 mM or 1 M NaCl. *Elution:* Immunoprecipitated proteins were eluted in LDS loading buffer (Invitrogen) containing 100 mM DTT by heating the beads for 10 minutes at 70 °C and separated on a NuPAGE 4–12% Bis-Tris protein gel (Invitrogen) using MOPS SDS running buffer (Invitrogen). Proteins were blotted to polyvinylidene fluoride (PVDF) membranes (Immobilon, Millipore).

### Western blot analysis

Membranes were blocked and incubated with primary antibodies against, GFP (ab290, Abcam), PAR (ab14459), PARP1 (sc-8002, Santa Cruz) or RNF146 (75–233, NeuroMab) before incubation with fluorescently labelled secondary antibodies (LI-COR Bioscience). Images were captured using Odyssey infrared imaging system (LI-COR Bioscience) and quantified using Odyssey Image Studio V2.

### Mass spectrometry (MS) analysis

Bands on the M.W. area of XRCC1 were excised and submitted to in-gel tryptic digestion. Tryptic digests were dried out, resuspended in 0.1% formic acid and analysed on a Thermo Scientific Orbitrap Elite mass spectrometer coupled to an Easy-nLC 1000 UHPLC system (Thermo Scientific). Peptides were injected onto a Acclaim PepMap100 C-18 column (75 μm i.d. × 2 cm, C18, 5 μm, 100 Å) (Thermo Scientific) and further separated on a Acclaim PepMap RSLC Nanoviper C-18 analytical column (50 μm i.d. × 15 cm, C18, 2 μm, 100 Å) (Thermo Scientific). An 80-minute method with a 55 minutes’ gradient starting with 100% Buffer A (0.1% Formic acid) with an increase in buffer B (100% Acetonitrile, 0.1% Formic acid) until 30% and a 250 nL/minute flow rate was employed. The peptides eluting from the column were analyzed in positive-ion mode under data dependent neutral loss MS3 mode using CID fragmentation with normalized collision energy 35. Each MS scan (m/z 400–1600) was acquired at a resolution of 120,000 FWHM, followed by 10 MS/MS scans triggered for intensities above 1.7E4. MS spectra were analyzed using Thermo Proteome Discoverer version 1.4.0.288 software running Mascot and the Sequest HT search algorithms. Spectra were searched against a Human RefSeq database with the following parameters: Max. missed cleavage = 2, precursor mass tolerance = 10 ppm, fragment mass tolerance = 0.05 Da, static modification: carbamidomethyl (C: +57.021 Da), dynamic modifications: Phospho (S,T,Y: +79.966), Oxidation (M: +15.995). Proteome Discoverer assign peptides identified with high degree of confidence as having False Discovery Rate (FDR) ≤ 0.01, medium confidence FDR ≤ 0.05, and low peptide confidence for FDR lower than 5%. The PhosphoRS 3.0 algorithm was also used to assign phosphorylations sites in XRCC1 and to calculate a site probability score, which determines confidence in phosphorylation site localization.

## Electronic supplementary material

Supplementary information
