# Supplementary material for: p38 MAPK signaling and phosphorylations in the BRCT1 domain regulate XRCC1 recruitment to sites of DNA damage
Source: Sci Rep. 2017 Jul 24;7:6322. doi: 10.1038/s41598-017-06770-3 (PMC5524842; doi:10.1038/s41598-017-06770-3)
Supplement: Supplementary file 1 — Supplementary information [file 41598_2017_6770_MOESM1_ESM.pdf]

p38 MAPK signaling and phosphorylations in the BRCT1 domain regulate XRCC1 recruitment to sites of DNA damage

Mirta Mittelstedt Leal de Sousa, Karine Øian Bjørås, Audun Hanssen-Bauer, Karin Solvang-Garten, Marit Otterlei

Supplementary Figure S1

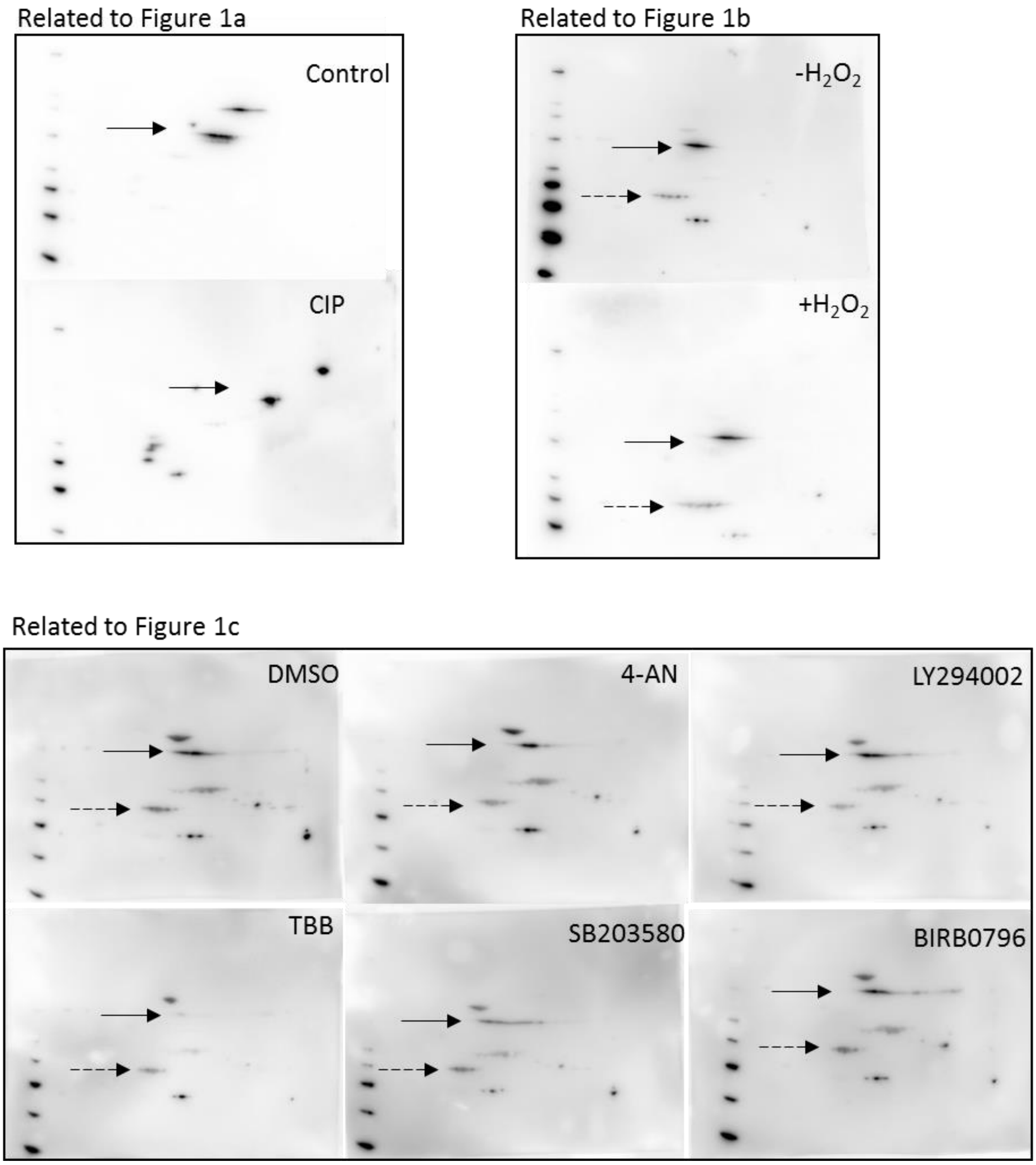

Supplementary Figure S1, related to Figure 1. 2D-PAGE western analysis of immunoprecipitated endogenous XRCC1 used in Figure 1. Full arrows indicate XRCC1 spots. IRF3 spots used as standards for alignment of XRCC1 spots are indicated by dotted arrows.

Supplementary Figure S2

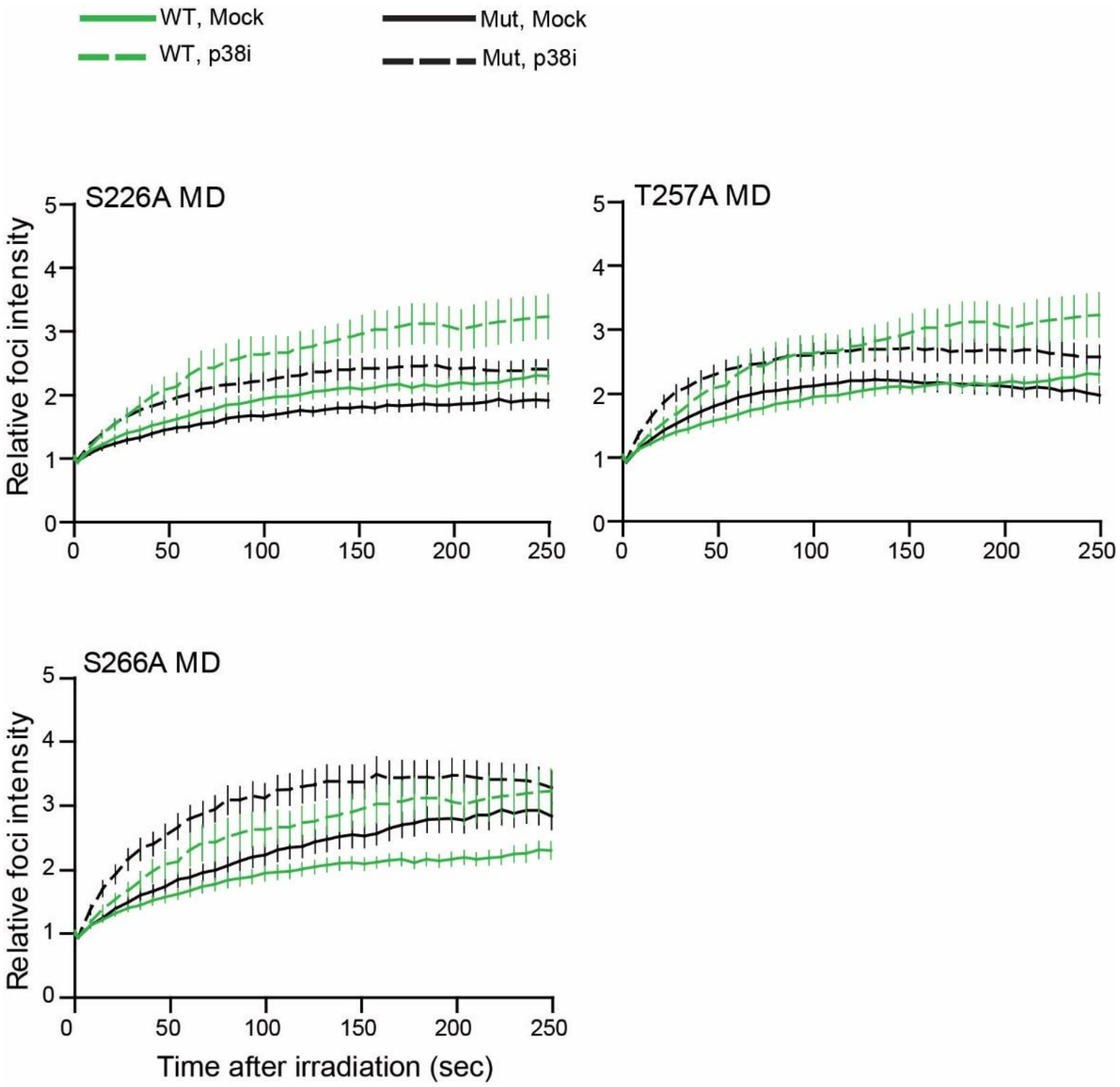

Supplementary Figure S2, related to Figure 3. Average fold increase in mIF intensities of wild type (green lines) and mutant (black lines) YFP-MD in mock and SB203580 (25 µM) treated CHO EM9 cells using 1x laser dose. WT: Mock n=19, p38i n=12, S226A: Mock n=18, p38i n=17. T257A: Mock n=17, p38i n=17. S266A: Mock n=17, p38i n=17.

Supplementary Figure S3

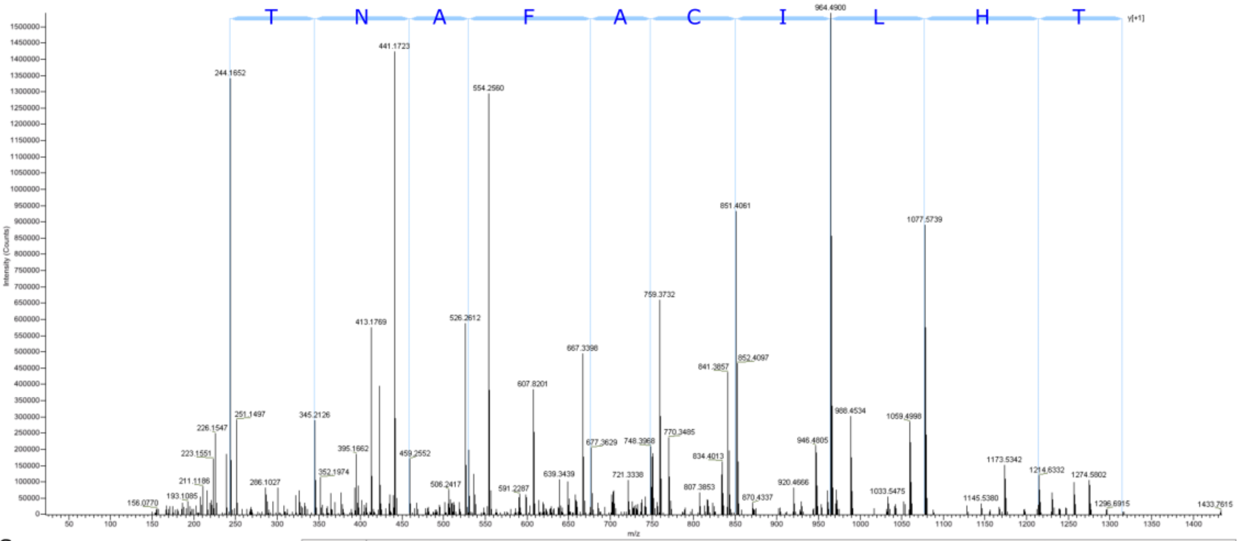

Sequence:
D **s** T H L I C A F A N T P K
Modification:
phospho S357 (79.96633 Da)

	(NH3+) -	s> <T	T> <H	H> <L	L> <I	I> <C	C>	A> <F	F> <A	A> <N	N> <T	T> <P	P> <K	K - (COOH)
b (+1)	116.03423	283.03259	384.08027	521.13918	634.22325	747.30732	850.31651	921.35363	1068.42205	1139.45917	1253.50210	1354.54978	1451.60255	1579.69752
b°(+1)	99.00768	266.00604	367.05372	504.11263	617.19670	730.28077	833.28996	904.32708	1051.39550	1122.43262	1236.47555	1337.52323	1434.57600	1562.67097
b*(+1)	98.02366	265.02202	366.06970	503.12861	616.21268	729.29675	832.30594	903.34306	1050.41148	1121.44860	1235.49153	1336.53921	1433.59198	1561.68695
y (+1)	1482.68113	1315.68277	1214.63509	1077.57618	964.49211	851.40804	748.39885	677.36173	530.29331	459.25619	345.21326	244.16558	147.11281	1597.70808
y°(+1)	1465.65458	1298.65622	1197.60854	1060.54963	947.46556	834.38149	731.37230	660.33518	513.26676	442.22964	328.18671	227.13903	130.08626	1580.68153
y*(+1)	1464.67057	1297.67221	1196.62453	1059.56562	946.48155	833.39748	730.38829	659.35117	512.28275	441.24563	327.20270	226.15502	129.10225	1579.69752

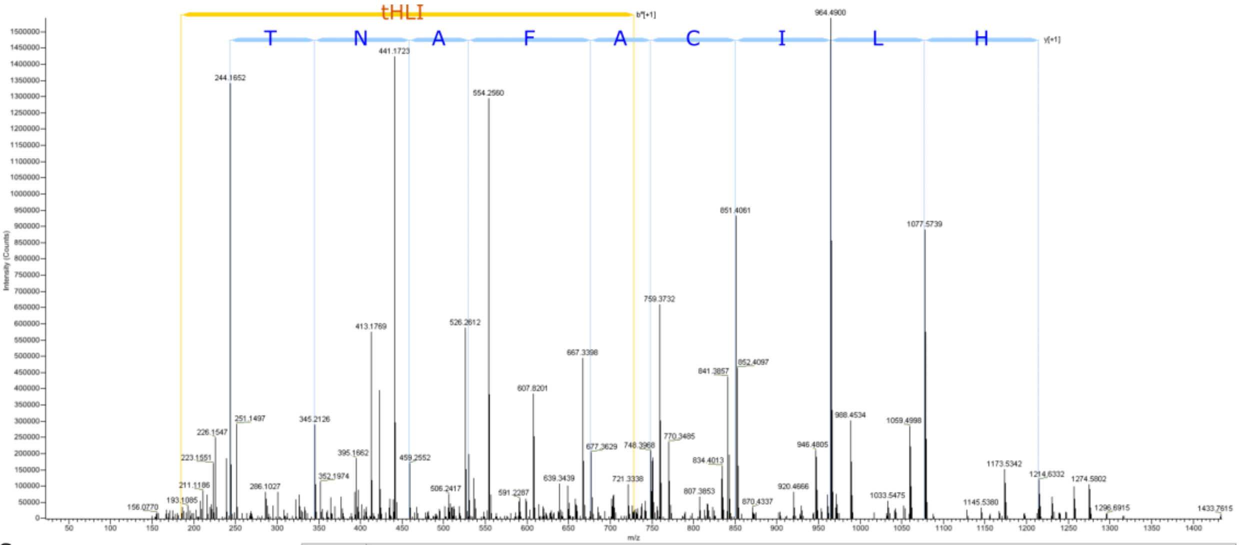

Sequence:
D S ***t*** H L I C A F A N T P K
Modification:
phospho T358 (79.96633 Da)

	(NH3+) -	S> <t	t> <H	H> <L	L> <I	I> <C	C>	A> <F	F> <A	A> <N	N> <T	T> <P	P> <K	K - (COOH)
b (+1)	116.03423	203.06626	384.08027	521.13918	634.22325	747.30732	850.31651	921.35363	1068.42205	1139.45917	1253.50210	1354.54978	1451.60255	1579.69752
b°(+1)	99.00768	186.03971	367.05372	504.11263	617.19670	730.28077	833.28996	904.32708	1051.39550	1122.43262	1236.47555	1337.52323	1434.57600	1562.67097
b*(+1)	98.02366	185.05569	366.06970	503.12861	616.21268	729.29675	832.30594	903.34306	1050.41148	1121.44860	1235.49153	1336.53921	1433.59198	1561.68695
y (+1)	1482.68113	1395.64910	1214.63509	1077.57618	964.49211	851.40804	748.39885	677.36173	530.29331	459.25619	345.21326	244.16558	147.11281	1597.70808
y°(+1)	1465.65458	1378.62255	1197.60854	1060.54963	947.46556	834.38149	731.37230	660.33518	513.26676	442.22964	328.18671	227.13903	130.08626	1580.68153
y*(+1)	1464.67057	1377.63854	1196.62453	1059.56562	946.48155	833.39748	730.38829	659.35117	512.28275	441.24563	327.20270	226.15502	129.10225	1579.69752

Supplementary Figure S3, related to Figure 4. Characterisation of phosphorylation sites in XRCC1 by LC-MS/MS. Phosphorylations on Serine 357 (top panel) and threonine 358 (bottom panel) were identified in Hela cells expressing XRCC1-YFP protein. Fragments corresponding to y-ions (blue) and b-ions (yellow) series identified for each spectrum are indicated.

Supplementary Figure S4

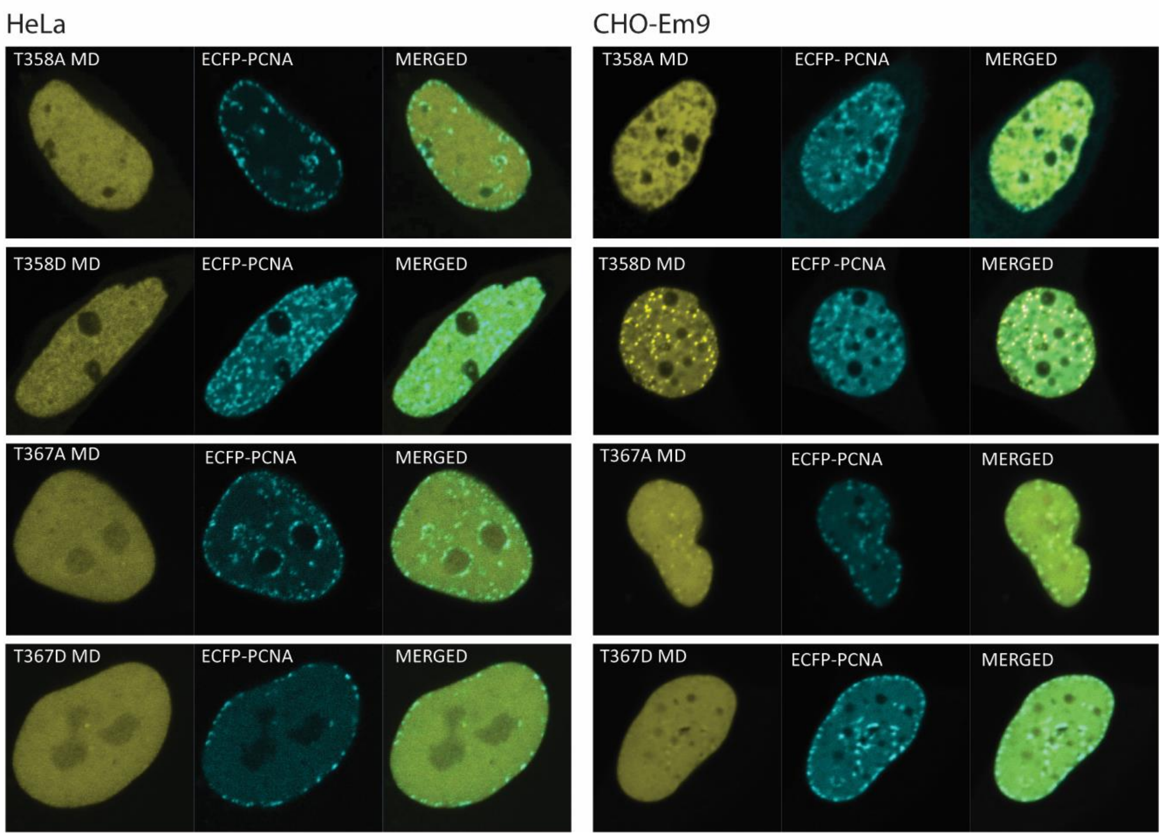

Supplementary Figure S4, related to Figure 5. Colocalization of YFP-MD T358A/D and T367A/D with CFP-PCNA in HeLa and CHO EM9 cells

Supplementary Figure S5

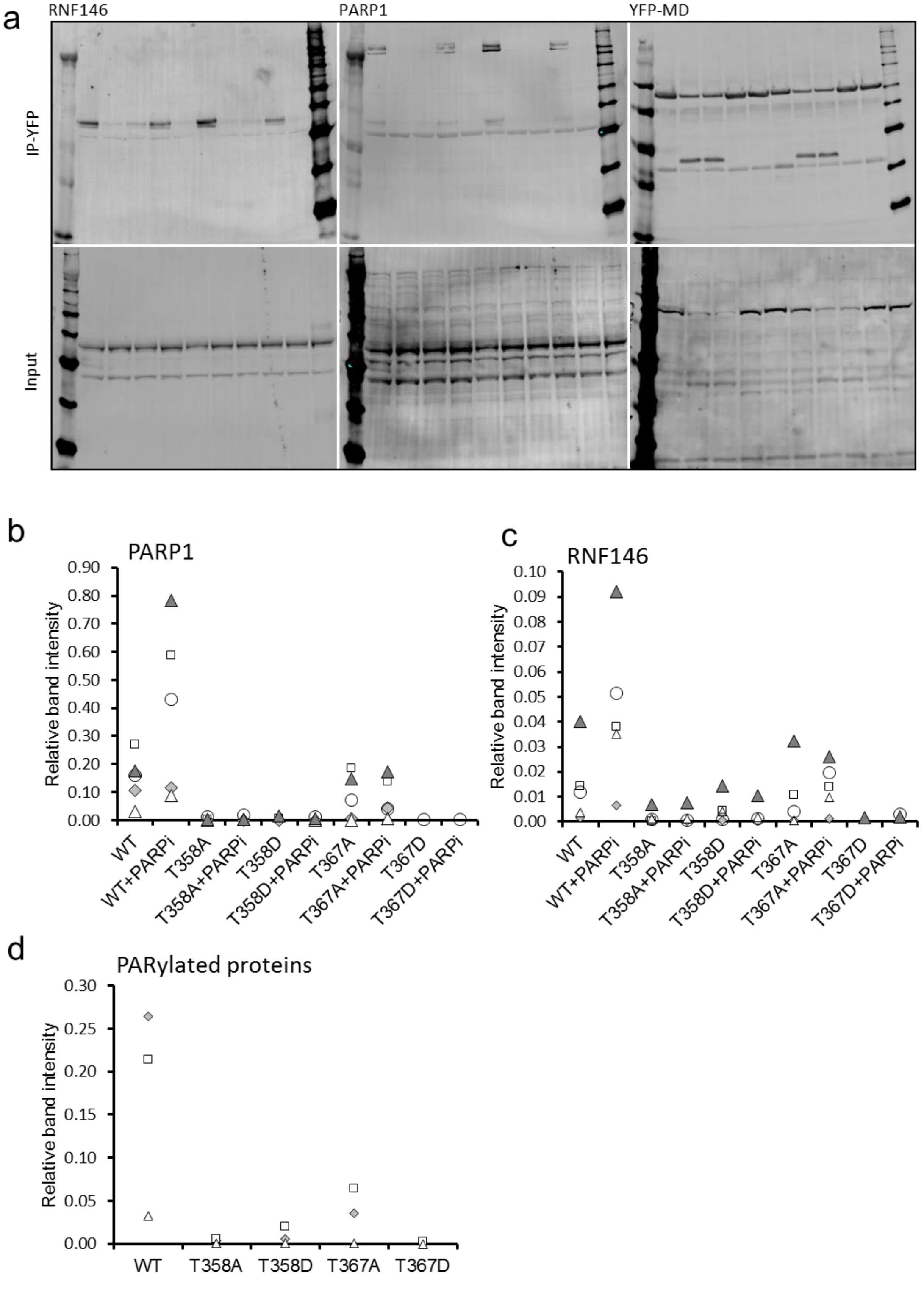

Supplementary Figure S5. Levels of PARP1, RNF146 and PARylated proteins co-immunoprecipitaded with YFP-MD mutants. **(a)** Original western blot images used in Figure 5. **(b)** PARP1 and **(c)** RNF146 relative band intensities (normalized against YFP-MD wt and mutants) in presence and absence of PARPi (10 µM PJ34) in five independent experiments. **(d)** Relative band intensities of PARylated proteins in three independent experiments.
